# Forced to stay at home—impact of curfews on mood during a pandemic for individuals with exercise dependence

**DOI:** 10.1007/s12662-022-00800-7

**Published:** 2022-02-25

**Authors:** Sinika Timme, Brian Cook, Melanie Schipfer, Oliver Stoll

**Affiliations:** 1 Sport and Exercise Psychology, University of Potsdam, Am Neuen Palais 10, 14469 Potsdam, Germany; 2 Alsana, Thousand Oaks, CA USA; 3 Institute of Sports Science, Martin-Luther-University Halle-Wittenberg, Von-Seckendorff-Platz 2, 06120 Halle (Saale), Germany

**Keywords:** Exercise addiction, COVID-19, Coronavirus disease 2019, Exercise behavior, Psychpathology

## Abstract

The effects of COVID-19-related lockdowns on deterioration of mental health and use of exercise to remediate such effects has been well documented in numerous populations. However, it remains unknown how lockdown restrictions impacted individuals differently and who was more likely to change their exercise behavior and experience negative well-being. The current study examined exercise dependence as a risk factor and its impact on exercise behavior and mood during the initial COVID-19 lockdowns on a global scale in 11,898 participants from 17 countries. Mixed effects models revealed that reducing exercise behavior was associated with a stronger decrease in mood for individuals at risk of exercise dependence compared to individuals at low risk of exercise dependence. Participants at high risk and exercising more prior to the pandemic reported the most exercise during lockdown. Effects of lowered mood were most pronounced in participants with high risk of exercise dependence who reported greater reduction in exercise frequency during lockdown. These results support recent etiological evidence for exercise dependence and add to a growing body of literature documenting mental health effects related to COVID-19.

## Introduction

There has been previous research suggesting that situations like the coronavirus disease 2019 (COVID-19) pandemic amplify addictive behavior. Brand, Young, Laier, Wölfling, and Potenza (2016) introduced the Interaction of Person-Affect-Cognition-Execution (I-PACE) model, which proposes that behavioral addictions are considered to develop as a consequence of interactions between predisposing variables (i.e., neurobiological and psychological constitutions), moderating variables (e.g., coping strategies) and mediating variables, such as affective and cognitive reactions to situational triggers in combination with reduced inhibitory control. As a result of conditioning processes, these associations become stronger within the addiction process. This is especially likely in the times of a pandemic, as individuals are faced with situations of less perceived control while coping resources, such as social gatherings or access to fitness centers, are severely limited.

The most current model is the expanded interactional model of exercise addiction (Dinardi, Egorov, & Szabo, 2021). This model is based on interactional model of exercise addiction, which describes its etiology (Egorov & Szabo, 2013). This model describes only primary exercise addiction which develops in the absence of eating disorders in contrast to secondary exercise addiction, in which certain eating disorders (i.e., anorexia, bulimia) are always present. Dinardi et al. (2021) added a new domain of ‘exercise-related stressors’ to the model. This domain interacts with ‘sudden or progressively intolerable stress’. By including this concept, they included a significant implication of exercising. Regardless of whether a person’s exercise is healthy or driven by addictive behavior, there is potential for them to experience stress due to their involvement with physical activity. For individuals who use exercise to escape from stress, this may result in spiraling further into a pattern of avoidance and escape, creating a dangerous circular causal relationship between physical activity and stress.

Effects of the COVID-19 pandemic have extended well beyond individuals who have been infected with the virus itself. It is clear that virus mitigation efforts, perceived risk of infection, and misinformation or evolving understanding of COVID-19 are associated with deterioration of mental health (e.g., negative mood, anxiety, depression; Holmes et al., 2020). Exercise has been one strategy to counter such effects (Castellini et al., 2020). Recent research demonstrated that a decrease in exercise was generally associated with more negative mood states (Brand, Timme, & Nosrat, 2020) but with substantial interindividual differences. Thus, it remains unclear who is specifically prone to changes in exercise behavior and possible effects on mood under these circumstances.

The combination of increased fear (Schimmenti, Billieux, & Starcevic, 2020) and performing exercise specifically to alleviate such negative affect (Castellini et al., 2020) raises concerns that individuals with exercise pathology would be at a particularly increased risk of symptom escalation during a pandemic. Exercise dependence (ExDep) is a proposed condition in which individuals engage in pathological patterns of exercise (Hausenblas & Symons Downs, 2002). Ecological momentary assessment studies have demonstrated that individuals with pathological exercise attitudes may regulate mood and affect through exercise behavior (Cook et al., 2013; Le Page, Price, O’Neil, & Crowther, 2012).

Moreover, recent etiological research suggests using exercise to regulate affect is a central aspect of pathological exercise (Kun et al., 2021). Thus, COVID-19-related amelioration efforts (e.g., lockdowns, stay-at-home orders, closing fitness centers) created an environment whereby access to typical exercise routines were greatly impacted and therefore may elicit psychological changes in individuals with ExDep.

The purpose of this study was to examine the effect of COVID-19 amelioration efforts on individuals with ExDep compared to individuals with no signs of ExDep. Specifically, we were interested in the change in exercise behavior by ExDep risk status and the relationship among ExDep and mood during lockdown restrictions.

## Methods

This study used a retrospective, cross-sectional design to investigate ExDep during COVID-19-specific lockdowns in an international sample. This is a secondary analysis of the data gathered of the “International Research Group (IRG) on COVID and exercise” (Brand et al., 2020). Surveys were completed during periods of COVID-19 lockdown restriction between March 29, 2020 and May 13, 2020.

### Participants and procedure

Participants were recruited by IRG members’ personal networks and press releases. The questionnaire was available in Arabic, Traditional and Simplified Chinese, Mandarin Chinese, English, Farsi, Filipino, Finnish, French, German, Greek, Icelandic, Italian, Malayan, Polish, Portuguese, Russian, Spanish, and Turkish. A total of 16,137 people completed the questionnaire. Individuals with incomplete data and self-reported symptoms of COVID-19 were excluded. In addition, only data from countries with more than 100 participants were considered for the time the country-specific lockdown restrictions were still in place (17 countries). The final sample included 11,898 (*M*_age_ = 33.94, *SD*_age_ = 13.73, 60.0% female) participants for the current study.

### Measures

#### Exercise behavior

Participants indicated number of days per week of exercise before (“How often did you exercise in the weeks before COVID-19?”) and during lockdown (“How often have you exercised lately (during COVID-19)?”) on a 9-point rating scale. Exercise was defined as any physical activity participants freely chose during their leisure time (e.g., workouts at home, running). Responses were grouped as *1 day or less, 2–3 days*, and *4 days or more *per week. Research has shown that single item measures of exercise behavior have reasonable validity (Prochaska, Sallis, & Long, 2001).

#### Exercise dependence

We used the 16-item FESA (Fragebogen zur Erfassung des Sportverhaltens bei Ausdauersportlern; Schipfer, 2015) to measure ExDep. The FESA yields subscales on exercise-related ‘expected positive consequences’, ‘interference with social life’, ‘health’, ‘withdrawal symptoms’, and ‘exercise as a possibility to compensate for psychological problems’ and an overall score. The FESA is a brief measure that can dynamically assess core aspects of ExDep and recent theoretical reviews support its use in community samples (Ziemainz, 2021). Internal consistency in the current study was satisfactory (α = 0.71).

#### Mood

Mood was assessed with the validated 16-item version of the Profile of Mood Scale (POMS; Petrowski, Albani, Zenger, Brähler, & Schmalbach, 2021). Participants were asked whether they experienced the respective feeling “not at all,” “a little,” “moderately,” “quite a lot” or “extremely” now and/or in the last few days during COVID-19. Higher scores indicate positive mood. Internal consistency in the current study was excellent (α = 0.89).

#### Statistical analysis

Cumulative link mixed models were used to examine the change in exercise behavior depending on individuals’ risk of ExDep. A linear mixed model was used to predict the influence of exercise behavior and ‘risk of ExDep’ on mood. The variable ‘country’ was included as a random effect in both mixed models. Mixed models were used to control for the hierarchical data structure (i.e., participants nested in countries) as it was assumed that individuals change their behavior differently due to different restrictions in the countries.

## Results

Overall, participants reported a mean score of 20.45 on the FESA (*SD* = 4.78, range: 6.25–38). 12.1% had a value above 26.2, indicating high risk of ExDep.

Exercise behavior prepandemic was a stronger predictor for risk of ExDep than exercise behavior during lockdown. While exercising more prepandemic increased the odds of being at high risk of ExDep (*OR*_bef:2‑1_ = 2.08, 95% CI [1.59, 2.77], *p* < 0.001; *OR*_bef:3‑2_ = 2.09, 95% CI [1.75, 2.50], *p* < 0.001), exercising more during lockdown increased the odds to a smaller degree (*OR*_dur:2‑1_ = 1.09, 95% CI [0.83, 1.42], *p* = 0.53; *OR*_dur:3‑2_ = 1.41, 95% CI [1.11, 1.81], *p* < 0.01).

### ExDep and exercise behavior

There was a significant effect for exercise frequency prepandemic and risk of ExDep. Thus, exercising more prepandemic (*OR*_bef:2‑1_ = 2.28, 95% CI [1.92, 2.71], *p* < 0.001; *OR*_bef:3‑2_ = 3.39, 95% CI [2.93, 3.92], *p* < 0.001) and ExDep risk (*OR*_high-low_ = 1.52, 95% CI [1.27, 1.82], *p* < 0.001) significantly increased the odds of exercising more during the lockdown.

### ExDep, exercise behavior and mood

The model with exercise frequency during lockdown as random slopes provided the best model fit (AIC = 22,312), taking into account the significant variability in the relationship between mood and exercise frequency during lockdown among countries (χ^2^(5) = 25.11, *p* < 0.001).

Predicting mood with exercise frequency prepandemic, during lockdown and ExDep revealed exercising more during lockdown (*b*_dur:2‑1_ = 0.18, *p* < 0.001, *b*_dur:3‑2_ = 0.12, *p* < 0.001) and low risk of ExDep was generally associated with higher mood (*b*_risk:low-high_ = 0.29, *p* < 0.001). However, there were significant interaction effects for exercise frequency and risk of ExDep, indicating that the relationship between exercise behavior and mood was different depending on the risk of ExDep.

**Fig. 1:**
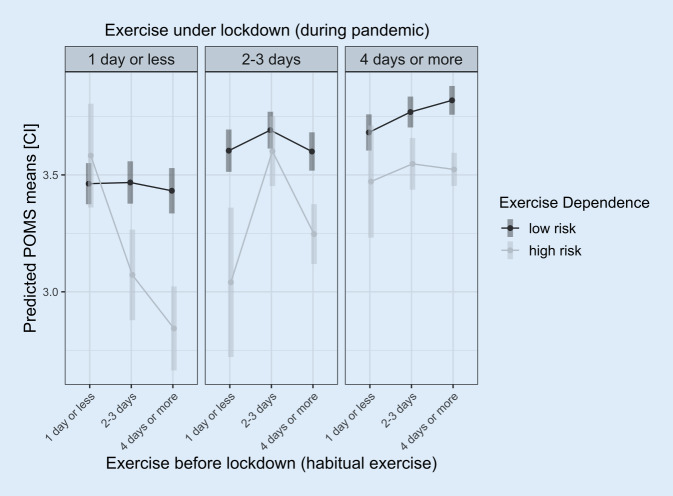
Mood by exercise dependence groups and exercise frequency

Reducing exercise behavior was associated with a stronger decrease in mood for individuals with high risk of ExDep compared to individuals with low risk of ExDep (Fig. 1). Specifically, reducing exercise frequency when being highly active prepandemic (*4 days or more*) was associated with a significantly lower mood for those with high risk of ExDep compared to those with a low risk of ExDep (*p* < 0.001; *b*_low:1-high:1_ = 0.68, *p* < 0.001; *b*_low:2 vs. high:2_ = 0.28,) (Fig. 1).

## Discussion

This study was among the first to examine associations among mood, exercise behavior and ExDep symptoms in a large multinational sample when exercise opportunities were greatly reduced due to widespread societal lockdowns. Individuals at risk of ExDep experienced more negative moods during lockdown. Specifically, reduced exercise behavior was related to increased negative moods. Thus, this study supports recent work suggesting managing negative emotions play an etiological role in ExDep (Kun et al., 2021).

Our observation of associations among mood and ExDep is in line with initial observations of exercise deprivation effects on habitual exercisers (Hausenblas & Symons Downs, 2002) and the etiological role of affect regulation in ExDep (Cook, Hausenblas, & Freimuth, 2014). In general, participants were most likely to maintain their habitual exercise behavior during the lockdown. However, this was different for individuals at risk of ExDep. Especially inactive participants prepandemic were more likely to increase their exercise behavior during lockdown. These results contrast slightly with recent reviews (e.g., Stockwell et al., 2021) which indicate that individuals in the general population were more likely to decrease their exercise behavior during COVID-19-related restrictions. However, samples with higher risk of ExDep (Cook et al., 2014) have been observed to increase their exercise behavior during initial phases of a pandemic (Castellini et al., 2020).

Results from the current study are in line with ecological momentary assessment studies suggesting exercise might be used as a compensation strategy of individuals with pathological exercise attitudes to regulate negative affect (Cook et al., 2014). Therefore, decreased mood may be a potential explanation for why high-risk participants were more likely to increase their exercise behavior, even when their usual exercise opportunities were removed. However, our study design could not determine whether lack of exercise resources due to lockdown were the cause for decreased mood. Moreover, individuals who did not maintain or reduced their exercise behavior were at high risk of experiencing negative mood. Thus, these data suggest that individuals at risk of ExDep may be likely to experience negative moods due to the alteration in routine activities.

There are some limitations inherent in the current study that must be taken in context when interpreting these data. The self-report retrospective cross-sectional design was necessary to collect data from a large international sample of participants during the limited time of lockdowns. However, this design is descriptive and therefore precludes the ability to infer causality. Thus, it remains possible that our finding of increases in positive mood may be a result of exercise behavior used as an emotional regulatory strategy. Further research is needed to examine the emotional valence of exercise in individuals with ExDep compared to exercisers without ExDep. Second, the scope of the current study was not able to differentiate between primary or secondary ExDep or other etiological factors implicated in ExDep development and maintenance. In addition, the use of the FESA as our measurement of ExDep may be viewed as a limitation of the current study because this measure was validated on endurance athletes. However, the FESA is not contraindicated in non-endurance samples because it assesses core aspects of ExDep (Cook et al., 2014; Hausenblas & Symons Downs, 2002). Specifically, all factors in the FESA correspond to definitions of behavioral addiction and reflect factors assessed in other measures of ExDep that have been used in population-based research. Finally, a recent population-based international study on the effect of COVID-19 on ExDep found support for the conceptual model upon which the FESA was derived (de la Vega et al., 2020).

The period of lockdown restrictions stemming from COVID-19 offered a unique opportunity to examine suppositions about the effect of ExDep on mood. Our results suggesting differences in mood are influenced by exercise history and ExDep status support recent etiological research (Kun et al., 2021). These results offer insights into the etiology of ExDep as a maladaptive coping strategy, potential behavioral addiction, and may guide future research examining the complex relationships between exercise behavior, mood, and ExDep.

## Declarations

For this article no studies with human participants or animals were performed by any of the authors. All studies mentioned were in accordance with the ethical standards indicated in each case.
